# Pro-inflammatory cytokines as potential predictors for intradialytic hypotension

**DOI:** 10.1080/0886022X.2021.1871921

**Published:** 2021-01-17

**Authors:** Jinbo Yu, Xiaohong Chen, Yang Li, Yaqiong Wang, Xuesen Cao, Zhonghua Liu, Bo Shen, Jianzhou Zou, Xiaoqiang Ding

**Affiliations:** a Division of Nephrology, Zhongshan Hospital, Shanghai Medical College, Fudan University, Shanghai, PR China; b Shanghai Institute of Kidney Disease and Dialysis, Shanghai, PR China; c Shanghai Key Laboratory of Kidney and Blood Purification, Shanghai, PR China

**Keywords:** Intradialytic hypotension, pro-inflammatory cytokines, tumor necrosis factor-α, interleukin-1β, hemodialysis

## Abstract

**Background:**

Intradialytic hypotension (IDH) is a common complication in maintaining hemodialysis (MHD) patients. Immune activation might be part of the mechanisms. However, the association between pro-inflammatory cytokines and blood pressure (BP) has not been deeply explored. So we aim to evaluate the potential role of pro-inflammatory cytokines in IDH.

**Methods:**

MHD patients starting hemodialysis before January 2016 were enrolled in our retrospective study. Patients' characteristics, laboratory results, and intradialytic BP were collected. IDH was defined as nadir systolic BP ≤ 90 mmHg during hemodialysis. The definition of IDH group was that those who suffered from more than one hypotensive event during one month after the enrollment (10% of dialysis treatments). Spearman correlation analysis and logistic regression were employed to explore the relationship between pro-inflammatory cytokines and IDH.

**Results:**

Among 390 patients, 72 were identified with IDH (18.5%). High levels of serum tumor necrosis factor-α (TNF-α) and interleukin-1β (IL-1β) were observed in the IDH group (*p* < 0.001). Both TNF-α and IL-1β positively correlated with predialysis BP (*p* < 0.01). Receiver operating characteristic curve (ROC) analysis was used to evaluate the diagnostic accuracy of serum IL-1β and TNF-α for IDH. The area under the curve of IL-1β was 0.772 (95% CI: 0.708-0.836, *p* < 0.01), and that of TNF-α was 0.701 (95% CI: 0.620-0.781, *p* < 0.01). After adjusting for patients' characteristics, biochemical parameters, comorbid conditions, predialysis BP, and medications, elevated TNF-α and IL-1β were still risk factors for IDH.

**Conclusion:**

Pro-inflammatory cytokines (TNF-α and IL-1β) could be potential predictors for IDH.

## Introduction

Intradialytic hypotension (IDH) is one of the most common complications in maintenance hemodialysis (MHD) patients and it occurs about 5% and 30% based on various definitions among different populations [1]. IDH affects not only the conventional hemodialysis procedures but also severely affects patients’ quality of life and prognosis [2,3]. Both blood pressure (BP) decline and associated tissue perfusion changes are associated with short-term or long-term vital organ complications [4–6]. Common risk factors for IDH may counteract hypovolemia during dialysis by triggering cardiovascular hemodynamic mechanisms.

Inflammatory markers are usually elevated in chronic kidney disease (CKD), and their levels seem to be related to malnutrition, maintenance of residual renal function, and volume status. Nevertheless, the mechanisms of chronic immune activation in MHD patients are not explored. Blood exposure to dialysis membranes, non-sterile dialysate use, retention of cytokines, acidosis, and infections are potential causes of immune activation in MHD patients [7,8]. Increased inflammatory markers are associated with volume overload and hypoalbuminemia in hemodialysis patients [9,10]. Gut hypo-perfusion induced by IDH might increase systemic endotoxin level. Endotoxemia is associated with chronic inflammation and cardiovascular risk by increasing proinflammatory cytokine production, oxidative stress and endothelial dysfunction [11]. In a word, IDH leads to hemodynamic instability, and repetitive hemodynamic instability may contribute to chronic inflammation. It might suggest that the frequent onset of IDH could lead to elevated serum inflammatory markers.

Cytokines may lead to inflammation and are thought to play an essential role in dialysis-related morbidity. One hypothesis is that the release of pro-inflammatory cytokines, such as tumor necrosis factor - α (TNF - α) and interleukin-1β (IL-1β), play a role in the occurrence of hemodialysis related acute symptoms such as fever and hypotension [12]. However, the relationship between inflammatory markers and intradialytic BP, especially IDH, has significantly remained unexplored.

Therefore, our study aims to explore the potential association between pro-inflammatory cytokines and IDH. We hypothesized that high predialysis pro-inflammatory cytokines might be potential predictors for IDH.

## Materials and methods

### Patients

Patients who started hemodialysis before January 2016 in the blood purification center, Zhongshan Hospital, Fudan University, Shanghai, China, were enrolled in our study. Inclusion criteria were: ① Patients older than 18, ② undergoing dialysis three times a week for at least three months, ③ in a stable condition. Patients with malignant tumor, active infection, autoimmune disease, liver dysfunction, cardiac dysfunction, and immunosuppressive agents three months before the study were excluded. The study was approved by our institutional clinical research ethics review board (Ethics Committee of Zhongshan Hospital, Fudan University) and was conducted according to the Declaration of Helsinki principles. Informed consent was signed by each patient before entering the study.

### Hemodialysis protocol

As mentioned elsewhere, our blood purification center is in a pioneer position in China [13]. Patients were treated with a 1.4 m^2^ synthetic membranes dialyzer (BLS514SD, Sorin Group Italia, Mirandola, Italy; Poly-flux14L, Gambro Dialysatoren GmbH, Hechigen, Germany) for low-flux HD. Standard bicarbonate dialysate (Na^+^: 138.0 mmol/L, K^+^: 2.0 mmol/L, Ca^2+^: 1.25 mmol/L, Mg^2+^: 0.5 mmol/L, HCO_3_^−^: 32.0 mmol/L) was used. Dialysate flow rate was 500 mL/min, and blood flow rate was 200 to 280 mL/min [14]. Dialysis water quality was checked monthly, meeting the standards of the association for the Advancement of Medical Instrumentation.

### Date collection and biochemical measurements

At the beginning of the study, physicians collected information about demographics, comorbidity, biochemistry, and antihypertensive medications.

Laboratory parameters, including cytokines (TNF-α, IL-1β, IL-2R, IL-6, IL-8, IL-10), were measured in the routine clinical laboratory. In our hospital, cytokines were measured by chemiluminescence quantitative measurement on IMMULITE 1000 analyzer using IMMULITE 1000 Kits (Siemens Healthcare Diagnostics, UK). The reference value of serum TNF-α and IL-1β level in our hospital was <8.1 and <5.0 pg/mL.

### BP measurements, IDH definition, and patient group

Peridialytic BP data were collected one month after the enrollment. Before each treatment, BP was taken in a sitting position according to the standard protocol, used an automatic stand-alone device or an integrated device in the dialysis machine, and had a suitable pressure cuff around the non-access upper arm, located at the heart level [15]. Post-dialysis BP was taken just before detaching the dialysis circuit from the patient following the same protocol. Peridialytic BPs were estimated by averaging all the BPs monitored every dialysis session for 12 consecutive routines after the study initiation.

IDH was defined as nadir systolic BP (SBP) ≤90 mmHg during hemodialysis [2]. The definition for IDH group was that patients with more than one hypotensive event during one month (10% of dialysis treatments) [2,3,16].

### Statistical analysis

Continuous variables were expressed as mean ± Standard Deviation, while categorical variables were appropriately presented as numbers and percentages. Student t-test was used to compare normal variables, whereas, for categorical variables, chi-square tests were performed, respectively. Spearman correlation analysis was used to evaluate the association between serum cytokines (TNF-α, IL-1β, IL-2R, IL-6, IL-8, IL-10) and peridialytic BPs, including predialysis BP and post-dialysis BP. Receiver operating characteristic curve (ROC) and logistic regression analysis were employed to test the predictive effect of predialysis serum TNF-α and IL-1β on IDH. We constructed a series of models: (1) Model 1: adjusted for demographic data (age, sex, and body mass index) + dialysis information (interdialytic weight gain, residual renal function, dialysis vintage, and single-pool Kt/V); (2) Model 2: adjusted for model 1 + biochemical data (serum albumin, pre-albumin, creatinine, and hemoglobin); (3) Model 3: adjusted for model 2 + cardiac conditions (left ventricular ejection fraction) + comorbid conditions (history of coronary heart disease, primary hypertension, and diabetes); (4) Model 4: adjusted for model 3 + predialysis BP (predialysis systolic BP and diastolic BP); (5) Model 5: adjusted for model 4 + antihypertensive medications. Multivariate regression analysis was used to analyze the covariates with *p* < 0.05 in univariate regression. Two-tailed *p* < 0.05 was considered significant. All analyses were performed using SPSS 24 (SPSS, Chicago, IL, USA).

## Results

### Patient characteristics and the incidence of IDH

There were 390 patients (238 males and 152 females) enrolled, mean age 59.53 ± 14.34 years. 72 patients were with IDH (≥1 hypotensive event in one month), and 318 patients were with no-IDH (<1 hypotensive event in one month). Demographic, clinical, and biochemical variables are presented in Table 1. In the IDH group, higher ultrafiltration rate, predialysis BP, serum β_2_MG, TNF-α, IL-1β, and lower post-dialysis BP, hemoglobin were observed (*p* < 0.05). There were more patients comorbid with hypertension and diabetes mellitus in the IDH group than those in the no-IDH group (*p* < 0.05).

**Table 1.** Demographic, clinical and biochemical data of the patients.

Characteristics	All (*n* = 390)	no-IDH (*n* = 318)	IDH (*n* = 72)	*p* Value
Demographic data				
Age (year)	59.53 ± 14.34	59.37 ± 14.56	60.21 ± 13.43	0.655
Male (%)	238 (61%)	191 (60.1%)	47 (65.3%)	0.413
BMI (kg/m^2^)	22.73 ± 3.44	22.68 ± 3.42	22.94 ± 3.54	0.554
Dialysis information				
Normalized ultrafiltration rate (ml/h/kg)	10.42 ± 2.94	10.07 ± 2.94	11.71 ± 2.57	<0.001
IDWG, kg	2.35 ± 2.36	2.26 ± 2.59	2.67 ± 1.09	0.191
Preserved residual kidney function	13 (3.3%)	9 (2.8%)	4 (5.6%)	0.245
Duration of dialysis (months)	18.33 ± 8.28	18.99 ± 8.20	16.33 ± 8.39	0.193
Dry weight (kg)	60.59 ± 13.87	60.40 ± 13.93	61.32 ± 13.71	0.615
spKt/Vurea	1.22 ± 0.32	1.21 ± 0.28	1.23 ± 0.44	0.660
Peridialytic blood pressure				
Predialysis-SBP (mmHg)	138.59 ± 17.60	137.01 ± 17.73	143.62 ± 16.31	0.005
Predialysis-DBP (mmHg)	77.18 ± 10.04	76.25 ± 9.83	80.11 ± 10.21	0.004
Postdialysis-SBP (mmHg)	128.60 ± 19.86	132.33 ± 18.91	116.76 ± 18.18	<0.001
Postdialysis-DBP (mmHg)	75.75 ± 10.94	77.35 ± 10.52	70.67 ± 10.77	<0.001
Biochemical data				
Hemoglobin (g/L)	112.13 ± 16.47	116.85 ± 13.98	111.06 ± 16.82	0.007
Serum albumin (g/L)	36.75 ± 10.46	36.92 ± 10.21	36.06 ± 11.49	0.537
Serum prealbumin (g/L)	0.30 ± 0.14	0.30 ± 0.14	0.30 ± 0.15	0.882
Serum creatinine (μmol/L)	837.92 ± 416.17	836.70 ± 415.46	843.24 ± 422.10	0.905
iPTH (ng/L)	315.55 ± 267.74	309.55 ± 269.53	342.01 ± 259.90	0.354
Serum β_2_MG (mg/L)	37.86 ± 8.67	37.36 ± 8.76	40.03 ± 7.95	0.018
hs CRP (mg/L)	8.46 ± 15.03	8.24 ± 15.42	9.42 ± 13.28	0.548
EF (%)	59.44 ± 18.67	60.53 ± 17.72	56.06 ± 21.19	0.141
Inflammatory markers				
TNF-α, pg/ml	50.27 ± 51.65	45.84 ± 40.59	69.82 ± 82.38	<0.001
IL-1β, pg/ml	84.82 ± 94.38	71.88 ± 82.57	145.18 ± 120.45	<0.001
IL-2R, U/ml	1368.87 ± 487.42	1352.36 ± 456.60	1441.36 ± 602.85	0.162
IL-6, pg/ml	65.12 ± 128.76	63.18 ± 128.39	73.25 ± 130.93	0.557
IL-8, pg/ml	850.74 ± 1522.37	789.18 ± 1498.88	1128.60 ± 1606.10	0.091
IL-10, pg/ml	9.71 ± 6.86	8.77 ± 5.15	12.18 ± 10.50	0.361
Medications				
Antihypertensive medication type	2.15 ± 1.21	2.16 ± 1.23	2.11 ± 1.13	0.793
Antihypertensive medication dosage (tablets)	2.71 ± 1.87	2.75 ± 1.92	2.58 ± 1.72	0.486
Comorbidity				
Hypertension (%)	296 (75.9%)	226 (71.1%)	70 (97.2%)	0.020
Diabetes mellitus (%)	72 (18.5%)	48 (15.1%)	24 (33.3%)	0.009
Cardiovascular disease (%)	44 (11.3%)	34 (10.7%)	10 (13.9%)	0.907
Cerebrovascular disease (%)	27 (6.9%)	23 (7.2%)	4 (5.6%)	0.343

Values are mean (SD) for continuous variables and % (n) for categorical variables. *P*-value, IDH vs no-IDH. BMI, body mass index; IDWG, interdialytic weight gain; SBP, systolic blood pressure; DBP, diastolic blood pressure; iPTH, intact parathyroid hormone; β_2_MG, β_2_-microglobin; hs CRP, high sensitive C-reaction protein; EF, ejection fraction; TNF-α, tumor necrosis factor-α; IL-1β, interleukin-1β; IL-2R, interleukin-2 receptor; IL-6, interleukin-6; IL-8, interleukin-8; IL-10, interleukin-10.

### Inflammatory cytokines and peridialytic BP

Serum TNF-α and IL-1β were elevated in the IDH group (*p* < 0.001) (shown in Table 1). Therefore, we tested the correlation between inflammatory cytokines (TNF-α, IL-1β, IL-2R, IL-6, IL-8, IL-10) and peridialytic BP (predialysis BP and post-dialysis BP). Both TNF-α and IL-1β positively were found related to predialysis BP (*p* < 0.01) (Table 2).

**Table 2.** Correlation between Inflammatory markers and intradialytic blood pressure.

	preSBP	preDBP	postSBP	postDBP
TNF-α, pg/ml				
* r*	0.163**	0.169**	0.002	−0.026
* P*	0.005	0.004	0.976	0.653
IL-1β, pg/ml				
* r*	0.189**	0.250**	−0.059	−0.012
* P*	0.008	<0.001	0.409	0.865
IL-2R, U/ml				
* r*	0.037	−0.056	−0.009	−0.033
* P*	0.528	0.339	0.872	0.559
IL-6, pg/ml				
* r*	0.068	0.143*	0.025	−0.007
* P*	0.258	0.017	0.667	0.905
IL-8, pg/ml				
* r*	0.080	0.146*	−0.023	−0.024
* P*	0.168	0.011	0.681	0.669
IL-10, pg/ml				
* r*	0.215	−0.161	−0.238	−0.440
* P*	0.501	0.617	0.433	0.132

**p < 0.01, *p < 0.05.

TNF-α, tumor necrosis factor-α; IL-1β, interleukin-1β; IL-2R, interleukin-2 receptor; IL-6, interleukin-6; IL-8, interleukin-8; IL-10, interleukin-10; preSBP, predialysis systolic blood pressure; preDBP, predialysis diastolic blood pressure; postSBP, postdialysis systolic blood pressure; postDBP, postdialysis diastolic blood pressure.

### Inflammatory cytokines and intradialytic hypotension

**Figure 1.**
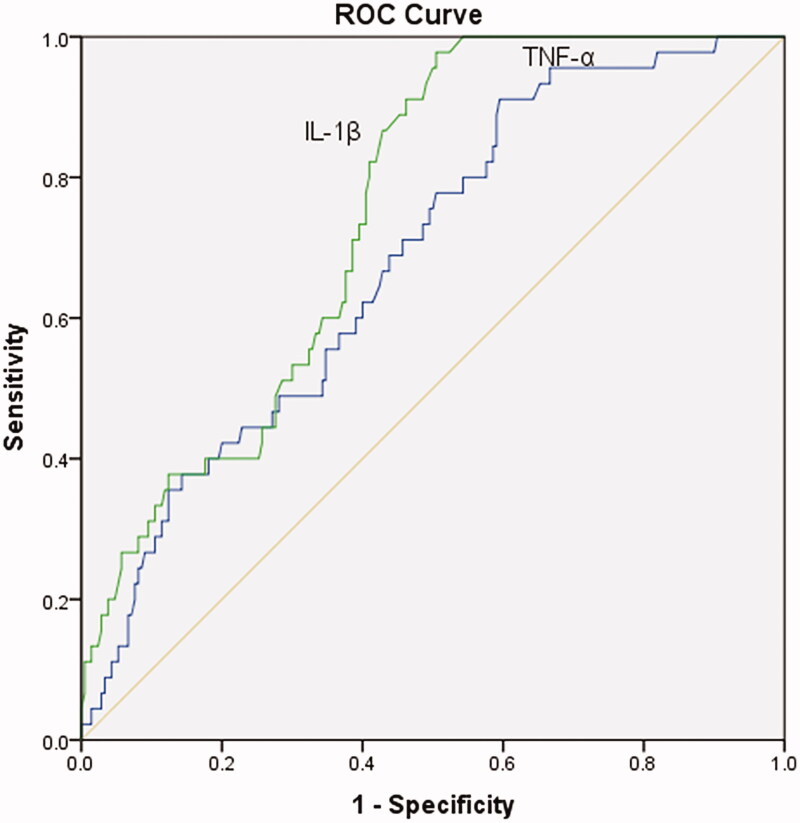
ROC curve of IL-1β and TNF-α for the onset of IDH.

Besides, ROC analysis was used to evaluate the diagnostic accuracy of serum IL-1β and TNF-α on IDH. The area under the curve of IL-1β was 0.772 (95% CI: 0.708-0.836, *p* < 0.01), and that of TNF-α was 0.701 (95% CI: 0.620–0.781, *p* < 0.01) (shown in Figure 1).

Furthermore, univariate and multivariate logistic regression models were used to investigate the influence of TNF-α and IL-1β on IDH. After adjusting for confounding factors like demographic data (age, sex, and body mass index), dialysis information (interdialytic weight gain, residual renal function, dialysis vintage and single-pool Kt/V), comorbid conditions (history of primary hypertension, coronary heart disease, and diabetes), biochemical data (serum albumin, pre-albumin, creatinine, and hemoglobin), cardiac conditions (left ventricular ejection fraction), predialysis BP (predialysis systolic BP and diastolic BP) and antihypertensive agents, predialysis serum TNF-α and IL-1β were still predictors for IDH (*p* < 0.05) (Table 3).

**Table 3.** Logistic regression analysis for predialysis serum IL-1β and TNF-α on intradialytic hypotension.

Models	serum IL-1β (per 1 pg/ml increase)		serum TNF-α (per 1 pg/ml increase)	
	*OR (95%CI)*	*p* Value	*OR (95%CI)*	*p* Value
Unadjusted	1.007 (1.004 ∼ 1.010)	<0.001	1.008 (1.003 ∼ 1.013)	0.003
Model 1	1.015 (1.006 ∼ 1.025)	0.002	1.014 (1.003 ∼ 1.025)	0.015
Model 2	1.015 (1.005 ∼ 1.025)	0.003	1.013 (1.002 ∼ 1.025)	0.024
Model 3	1.023 (1.006 ∼ 1.040)	0.008	1.015 (1.003 ∼ 1.027)	0.015
Model 4	1.031 (1.003 ∼ 1.061)	0.032	1.015 (1.002 ∼ 1.028)	0.028
Model 5	1.009 (1.000 ∼ 1.018)	0.039	1.014 (1.001 ∼ 1.028)	0.029

Model 1: adjusted for demographic data (age, sex, and body mass index) + dialysis information (interdialytic weight gain, residual renal function, dialysis vintage and single-pool Kt/V).

Model 2: adjusted for Model 1+ biochemical data (serum albumin, pre-albumin, creatinine and hemoglobin).

Model 3: adjusted for Model 2 + cardiac conditions (left ventricular ejection fraction) + comorbid conditions (history of primary hypertension, coronary heart disease, and diabetes).

Model 4: adjusted for Model 3 + predialysis blood pressure (predialysis systolic blood pressure and diastolic blood pressure).

Model 5: adjusted for Model 4 + antihypertensive medications.

## Discussion

We conducted this retrospective cohort study to evaluate the predictive effect of pro-inflammatory cytokines on IDH. Predialysis serum TNF-α and IL-1β were found positively correlated with predialysis BP. Serum TNF-α and IL-1β were predictors for IDH, and this finding was independent of a series of confounding factors.

IDH occurs when fluid removal rate exceeds plasma reperfusion rate and the related physiological compensation. The decrease of arterial blood volume caused by excessive ultrafiltration can decrease cardiac filling, cardiac output and hypotension [17]. So the risk factors of IDH are mainly determined by ultrafiltration rate (UFR) and compensatory ability. Compensatory mechanisms include enhanced cardiac output, plasma reperfusion, and vascular tension. Cardiac output is enhanced by increased contractility and heart rate. When the compensatory response of reduced cardiac filling reaches the limit, BP drops [18].

CKD is thought to be an inflammatory disease because many inflammatory stimuli could release cytokines such as IL-1, IL-6 and TNF-α in CKD patients. Signs of chronic and mild inflammation have appeared in the early stages of CKD [19]. Renal dysfunction may be linked with increased inflammatory response, as inflammatory cytokines and other inflammatory biomarkers elevated in mild [20] and severe [21] renal dysfunction. Because of the interaction between the dialysis membrane, dialysate, and the patient's blood, the initiation of dialysis treatment might enhance the inflammatory response [22,23]. However, studies showed no significant difference in the levels of serum IL-1 and TNF-α before dialysis initiation and after long-term treatment [24].

Association between fluid overload (FO) and inflammation was revealed in MHD patients [25–27]. Inflammation might theoretically contribute to FO because of hypoalbuminemia. Hypoalbuminemia can lead to vascular volume migration to the interstitial chamber, which hinders fluid removal during dialysis [28]. However, FO itself can also reduce serum albumin levels by dilution, as serum albumin levels increased after excessive ultrafiltration [29]. Inflammation may also result in interstitial fluid accumulation by increasing capillary permeability. FO may also bring about inflammation by endotoxin fragment passing through a congested intestinal wall or visceral ischemia [30,31]. As volume overload, hypoalbuminemia and malnutrition are common predictors of IDH. We presumed that inflammatory markers might play a part in the onset of IDH. Serum TNF-α and IL-1β were observed elevated in the IDH group (*p* < 0.001).

BP regulation partly depends on vasoactive agents as nitric oxide (NO) and endothelin-1 (ET-1). During dialysis, the activation of nitric oxide synthase (NOS) mediated by cytokines has been reported. The relationship between cytokine-mediated NO and ET-1 system activation and BP regulation in MHD patients has been investigated. Hypotension patients showed high NO end products, and hypertension patients had low levels of them, suggesting an influence of NO in BP control [32]. Other factors leading to endothelial dysfunction include the pro-inflammatory cytokines TNF-α and IL-1. Inflammatory cytokines have damaged NO-mediated vasodilatation through downregulating NOS mRNA levels and stimulating NOS [33,34]. In our study, predialysis serum TNF-α and IL-1β were positively related to predialysis BP.

There remains, in a way, the classical "chicken and egg" dilemma. It is not understood whether inflammation or intradialytic BP drops happens first. Hypotension and cardiovascular instability are the most common side effects of hemodialysis and have been connected with IL-1 and TNF-a synthesis in monocytes [22,23]. Previous studies have already focused on the role of NO in IDH [35]. The relationships between NO levels and dialysate and body temperatures were observed. NO synthetic capacity increased when dialysate temperature was 37.5 °C [36]. And core temperature increases with a dialysate temperature of 37.5 °C, bringing about cutaneous vasodilation to remove the excess heat [37,38]. It has been proposed that IDH may be caused by cytokine-induced NOS, an effective vasodilator in vascular smooth muscle cells and endothelial cells *in vitro* and *in vivo* [39]. A correlation between C-reactive protein (CRP) and IL-6 level and the change in BP was found in another study, suggesting that immune activation working through cytokines might contribute to the pathogenesis of IDH [39]. Inflammation, associated with hypoalbuminemia, leads to hypoperfusion during hemodialysis, making patients unable to achieve dry weight. Thus, extracellular fluid overload and endotoxin translocation might appear in the long-term, reversely enhancing inflammatory response. Likewise, hemodialysis, with excessive ultrafiltration, can lead to systemic severe hemodynamic disorders and perfusion reduction in vital organs [40]. Acute cardiac dysfunction, long-term myocardial injury, and increased mortality might be caused by repeated ischemic injury of vascular bed [41]. A previous study suggested that long-term MHD patients have evidence of mucosal ischemia [42], and ultrafiltration reduces visceral blood volume [43]. Mesenteric ischemia results in intestinal mucosa structure disorder and function and increases intestinal permeability [44]. In MHD patients, predialysis endotoxin was associated with hemodynamic instability (ultrafiltration, relative hypotension), myocardial stunning, and CRP. Also, endotoxemia, due to over ultrafiltration, is higher in the presence of hypervolemia and IDH [11]. In our study, we constructed a series of models to test the potential role of predialysis serum TNF-α and IL-1β in the onset of IDH. We found that both predialysis serum TNF-α and IL-1β could predict IDH.

In this study, we comprehensively assess the predictive role of predialysis serum TNF-α and IL-1β for IDH in MHD patients. We firstly testified the effect of pro-inflammatory cytokines on IDH in clinical practice. Moreover, we also confirmed the association of pro-inflammatory cytokines and peridialytic BP.

However, there are several limitations. One of these is that the study patients were stable, free of cardiac dysfunction. Future researches could target on MHD patients with various comorbidities. Another limitation is that the effect of different vascular accesses on hemodynamic stability was not assessed. Third, we did not use objective methods (e.g. bio-impedance) to evaluate fluid status. Fourth, we did not test pre-/post-dialytic NO levels due to NO's testing was still in the research lab, not like the cytokines readily available in clinical practice. Not evaluating NO levels might hinder us from exploring the underlying mechanisms. Moreover, we did not measure cytokine levels right after dialysis to assess the effect of hemodialysis sessions on serum cytokine concentrations and compare cytokine levels pre- and post-hemodialysis. The last important limitation is the nature of clinical researches, so the cause and effect relationship could not be specified. We have not dug further into the underlying mechanisms of the results. Future studies should focus on the possible mechanisms for the predictive effects of pro-inflammatory cytokines on IDH.

## Conclusions

Evidence has shown crosstalk between inflammation and IDH. IDH patients have elevated serum TNF-α and IL-1β levels. Pro-inflammatory markers are positively associated with predialysis BP. Pro-inflammatory cytokines (TNF-α and IL-1β) could be potential predictors of IDH. The preventive methods for IDH should involve traditional risk factors and consider inflammation effects since many pro-inflammatory markers are inexpensive, easy to measure, widely used, standardized, and might be included in the regular examination of patients at risk. Pro-inflammatory markers might help to predict IDH.
